# Comparative Performance of Current Patient-Accessible Artificial Intelligence Large Language Models in the Preoperative Education of Patients in Facial Aesthetic Surgery

**DOI:** 10.1093/asjof/ojae058

**Published:** 2024-08-13

**Authors:** Jad Abi-Rafeh, Brian Bassiri-Tehrani, Roy Kazan, Steven A Hanna, Jonathan Kanevsky, Foad Nahai

## Abstract

**Background:**

Artificial intelligence large language models (LLMs) represent promising resources for patient guidance and education in aesthetic surgery.

**Objectives:**

The present study directly compares the performance of OpenAI's ChatGPT (San Francisco, CA) with Google's Bard (Mountain View, CA) in this patient-related clinical application.

**Methods:**

Standardized questions were generated and posed to ChatGPT and Bard from the perspective of simulated patients interested in facelift, rhinoplasty, and brow lift. Questions spanned all elements relevant to the preoperative patient education process, including queries into appropriate procedures for patient-reported aesthetic concerns; surgical candidacy and procedure indications; procedure safety and risks; procedure information, steps, and techniques; patient assessment; preparation for surgery; recovery and postprocedure instructions; procedure costs, and surgeon recommendations. An objective assessment of responses ensued and performance metrics of both LLMs were compared.

**Results:**

ChatGPT scored 8.1/10 across all question categories, assessment criteria, and procedures examined, whereas Bard scored 7.4/10. Overall accuracy of information was scored at 6.7/10 ± 3.5 for ChatGPT and 6.5/10 ± 2.3 for Bard; comprehensiveness was scored as 6.6/10 ± 3.5 vs 6.3/10 ± 2.6; objectivity as 8.2/10 ± 1.0 vs 7.2/10 ± 0.8, safety as 8.8/10 ± 0.4 vs 7.8/10 ± 0.7, communication clarity as 9.3/10 ± 0.6 vs 8.5/10 ± 0.3, and acknowledgment of limitations as 8.9/10 ± 0.2 vs 8.1/10 ± 0.5, respectively. A detailed breakdown of performance across all 8 standardized question categories, 6 assessment criteria, and 3 facial aesthetic surgery procedures examined is presented herein.

**Conclusions:**

ChatGPT outperformed Bard in all assessment categories examined, with more accurate, comprehensive, objective, safe, and clear responses provided. Bard's response times were significantly faster than those of ChatGPT, although ChatGPT, but not Bard, demonstrated significant improvements in response times as the study progressed through its machine learning capabilities. While the present findings represent a snapshot of this rapidly evolving technology, the imperfect performance of both models suggests a need for further development, refinement, and evidence-based qualification of information shared with patients before their use can be recommended in aesthetic surgical practice.

**Level of Evidence: 5:**

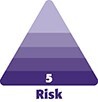

Artificial intelligence (AI) models represent computer systems that emulate human intelligence, leveraging autonomous perception, knowledge synthesis, and inference of information.^1,2^ Large language models (LLMs) are special AI programs with natural language processing capabilities that allow these models to solicit, interpret, and synthesize text in response to user commands.^3-7^

Of particular interest to our group is the role of AI in patient guidance and patient education. With a growing prevalence of Internet and social media use among patients in their self-directed research about their aesthetic and surgical needs,^8,9^ the role of AI and LLMs as patient resources cannot be ignored. Prior to adoption, it remains critical to objectively assess and validate the information these models share with our patients.^5^ The present study seeks to objectively evaluate and directly compare the performance of ChatGPT (OpenAI, San Fransisco, CA) and Bard (Google, Mountain View, CA), 2 novel and powerful LLMs freely available to patients, in answering questions from the perspective of simulated patients interested in facial aesthetic surgery. Questions posed spanned all elements in the preoperative patient education process, with a comparative assessment of responses performed using 6 objective assessment criteria. Identification of the model with superior performance will help guide the adoption, development, and regulation of AI in aesthetic surgical practice, with the goal of quality improvement and improved efficiency in aesthetic care delivery.

## METHODS

ChatGPT and Bard were queried through simulated interactions from the perspective of patients with different facial aesthetic surgical needs. Procedures targeted in the present study included facelift, rhinoplasty, and brow lift. Simulated questions were prepared and comprehensively covered all aspects of the preoperative patient education process, categorized under aesthetic needs inquiries and awareness of appropriate procedures; patient candidacy and procedure indications; procedure safety and risks; procedure information, steps, and techniques; patient assessment; preparation for surgery; recovery and postprocedure instructions; procedure costs; and recommendation of surgeons (Table 1). Three unique patient profiles were generated for each target procedure, with different lifestyle and medical risk factors (Supplemental Tables 1‐6). Questions for each procedure were identical and posed to both LLMs independently.

**Table 1.** Study Outline

Question category	Question posed
Aesthetic needs inquiry: awareness of available procedures	“I’m interested in getting facial plastic surgery. I’m bothered by (*concerns*). What are my options?”
Patient candidacy and procedure indications	“How do I know if I am a good candidate for (*procedure*)?”
Procedure safety and risks	“What are the risks associated with this procedure?”
	“Are there specific known estimates for each of these risks that you can share? And what is the overall complication rate of (*procedure*)?”
	“As a (*age and gender*), with (*medical and lifestyle risk factors*), am I a good candidate for (*procedure*)? Do the complication rates you shared apply to me, or am I at higher or lower risk?”
Procedure information, steps, and techniques	“Can you explain to me the steps of how a (*procedure*) is performed?”
	“Are there different techniques that surgeons use in (*procedure*)? Is one better than the other in terms of safety and overall results?”
Patient assessment	“If I were to upload a picture of myself, could you analyze my features and suggest which specific technique may be best for me?”
Preparation for surgery	“If I were to get a (*procedure*), what should I expect in the lead up to my surgery day?”
	“As a (*age and gender*) with (*medical and lifestyle risk factors*), is there anything I can do from now to prepare for this procedure, or make myself a better candidate?”
Recovery and postprocedural instructions	“What can I expect in terms of recovery after (*procedure*)?”
	“How long will I need assistance for after (*procedure*) with my day-to-day activities?”
	“How long do I have to wait after surgery before I can go out in public and not worry that people will know that I had (*procedure*)? How long before I can go back to work?”
Procedure cost and surgeon recommendations	“How much does a (*procedure*) cost? If I tell you where I live, are you able to recommend to me the best surgeons near me?”

Summary of standardized questions posed to ChatGPT and Bard to evaluate their performance in preoperative patient guidance and education in facial aesthetic surgery. Procedures examined included facelift, rhinoplasty, and brow lift.

All simulated interactions were performed by a single author (J.A.-R.) in a standardized and controlled environment during June 2023. The same computer (Apple MacBook Pro; Apple, Inc., CA), browser (Google Chrome; Google Inc., CA), and Internet connection were used. To eliminate the potential impact of locally stored data on the LLMs’ performance, all chats were closed, browser history (including cookies) cleared, and the computer restarted prior to each new session of interactions with the LLMs. Time required to provide each response, and response word counts, were documented.

An objective assessment of responses by the study's senior author (F.N.) followed. Assessment categories comprised accuracy of information shared; comprehensiveness; objectivity; safety; acknowledgment of limitations; and communication clarity. Likert scales ranging from 1 to 10 (with 10 representing perfect performance) were used, and responses from both LLMs were graded in tandem. Means and standard deviations were calculated using Microsoft Excel (Microsoft Technology Inc, Redmond, WA). Statistical analyses were performed using unpaired *t* tests, χ^2^ analysis, and 1-way analysis of variance, as appropriate, with a *P*-value <.05 taken to indicate statistical significance.

## RESULTS

### Performance Metrics

**Figure 1.**
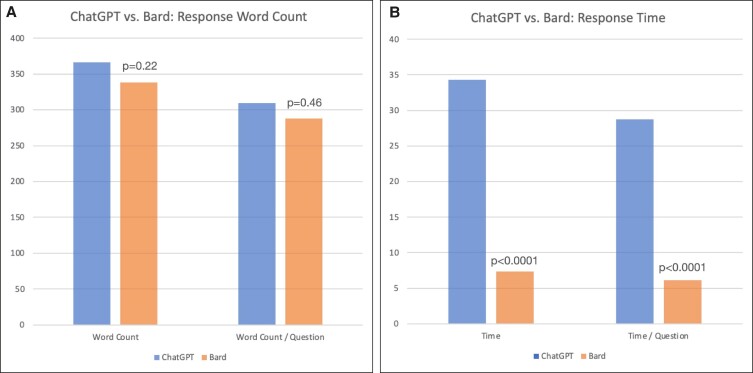
ChatGPT vs Bard average word counts and time. (A) Average response word counts and word counts per question (accounting for questions posed to the large language models [LLMs] with multiple subcomponents) for ChatGPT and Bard. No statistically significant differences were observed between both LLMs (367 ± 12 vs 338 ± 20 words and 309 ± 19 vs 288 ± 22 words, respectively). (B) Average response times and response time per question (accounting for questions posed to the LLMs with multiple subcomponents) for ChatGPT (blue) and Bard (orange). Bard response times were faster than those of ChatGPT (34.3 ± 1.7 vs 7.4 ± 0.2 s and 28.7 ± 2.0 vs 6.1 ± 0.3 s, respectively; *P* < .0001).

Average time required for ChatGPT to provide responses to posed questions was 34.3 s, with an average response length of 367 words (*n* = 42). When normalizing to the number of questions posed within each question stem (in cases of questions with multiple subcomponents), average response time was 28.7 s per question, with an average response length of 309 words per question. Average response time for Bard was significantly lower, at 7.4 s (*P* < .0001), while average response length was 338 words (*P* = .22). Normalized to the number of questions in each question stem, average response time was 6.1 s per question (*P* < .0001), and average response length was 288 words per question (*P* = .46, Figure 1, Table 2).

**Table 2.** Performance Metrics

Performance metrics	ChatGPT	Bard	Statistical analysis
Average response time	34.3 ± 1.7 s	7.4 ± 0.2 s	*P* < .0001
Average response time/question asked	28.7 ± 2.0 s	6.1 ± 0.3 s	*P* < .0001
Average response word count	367 ± 12 words	338 ± 20 words	*P* = 0.22
Average response word count/question asked	309 ± 19 words	288 ± 22 words	*P* = .46
Limitations acknowledgment/deferral to MD	*n* = 40/42 (95%)	*n* = 26/42 (62%)	*P* < .001
Sources provided in responses	*n* = 0/42 (0%)	*n* = 2/42 (5%)	NA
Response failures	*n* = 6/42 (14%)	*n* = 7/42 (17%)	*P* = .09

Summary of ChatGPT and Bard's overall performance metrics, with relevant statistical analyses. NA, not applicable.

**Figure 2.**
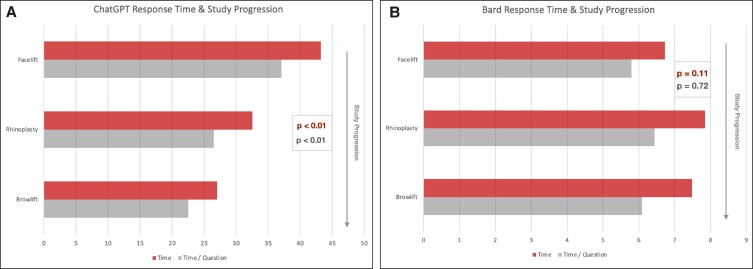
ChatGPT vs Bard reduction in response times with study progression. The study progressed with the facelift query, then rhinoplasty, then brow lift posed to both large language models (LLMs) in tandem. All chats were closed, browser history (including cookies) cleared, and computer restarted prior to each new session in order to eliminate any potential impact of locally stored data on the bot's efficiency and performance. ChatGPT (A), but not Bard (B), demonstrated a statistically significant reduction (*P* < .01) in response times with study progression, potentially reflecting the model's machine learning capabilities.

To assess the model's machine learning capabilities^2,10,11^ and potential impact of previous exposure to the same set of standardized questions on future performance, response times and word counts were compared in paired sets, across the order in which they were administered to the LLMs in this study, beginning initially with facelift, then rhinoplasty, then brow lift queries. No statistically significant differences in word counts were observed between ChatGPT and Bard as the study progressed (*P* = .83 and *P* = .60, respectively). Remarkably, a statistically significant reduction in response times was observed for ChatGPT, but not for Bard, indicating machine learning (*P* < .01 and *P* = .11, respectively; Figure 2).

### Disclaimers, Sources, and Response Failures

Overall, ChatGPT provided an explicit disclaimer regarding its limitations in answering medical questions as an AI model, deferred to a medical professional, or explicitly encouraged patients to consult with a surgeon in 95% of responses provided. In contrast, Bard provided such disclaimers in only 62% of its responses (*P* < .001). Sources were provided by Bard for responses in only 5% of cases, whereas all of ChatGPT's responses were devoid of references or sources. Response failures, representing the complete inability of an LLM to respond to the posed question, were observed in 17% of questions with Bard, and 14% with ChatGPT (*P* = .09, Table 2).

### Assessment

ChatGPT outperformed Bard in all assessment categories, and across all 3 facial aesthetic surgery procedures examined. ChatGPT obtained an overall assessment score of 8.1/10, whereas Bard scored 7.4/10. Overall accuracy of information was scored at 6.7/10 ± 3.5 for ChatGPT; comprehensiveness at 6.6/10 ± 3.5, objectivity at 8.2/10 ± 1.0, safety at 8.8/10 ± 0.4, communication clarity at 9.3/10 ± 0.6, and acknowledgment of limitations at 8.9/10 ± 0.2 (Table 3). With regards to specific procedures examined, ChatGPT's average score was 8.0/10 for facelift; 8.2/10 for rhinoplasty, and 8.0/10 for brow lift (Supplemental Tables 1-3). For Bard, overall, accuracy of information was scored at 6.5/10 ± 2.3; comprehensiveness at 6.3/10 ± 2.6, objectivity at 7.2/10 ± 0.8, safety at 7.8/10 ± 0.7, communication clarity at 8.5/10 ± 0.3, and acknowledgment of limitations at 8.1/10 ± 0.5 (Table 4). Bard's overall performance was scored at 7.2/10 for facelift, 7.7/10 for rhinoplasty, and 7.3/10 for brow lift (Supplemental Tables 4-6). A detailed breakdown of performance across all 8 standardized question categories, 6 assessment criteria, and 3 facial aesthetic surgery procedures examined is presented in Tables 3-5 and Supplemental Tables 1-6.

**Table 3.** ChatGPT Performance Assessment Scores Across All Simulated Patient Inquiries (Facelift, Rhinoplasty, and Brow Lift)

ChatGPT overall scores	Accuracy of information	Comprehensiveness	Objectivity of information	Safety of information shared with patient	Acknowledgment of limitations	Communication, clarity, patient-appropriate readability	Average
Aesthetic needs inquiry; awareness of available procedures	9.0 ± 0.0	9.0 ± 1.0	9.0 ± 0.0	9.0 ± 0.0	9.0 ± 0.0	10.0 ± 0.0	9.2 ± 0.4
Patient candidacy and procedure indications	9.0 ± 0.0	9.3 ± 0.6	9.0 ± 0.0	9.3 ± 0.6	9.0 ± 0.0	10.0 ± 0.0	9.3 ± 0.4
Procedure safety and risks	8.3 ± 0.6	8.0 ± 1.0	8.3 ± 0.6	8.7 ± 0.6	8.7 ± 0.6	9.0 ± 0.0	8.5 ± 0.3
Procedure information, steps, and techniques	8.3 ± 0.6	8.0 ± 1.0	8.7 ± 0.6	9.0 ± 0.0	9.0 ± 0.0	9.3 ± 0.6	8.7 ± 0.5
Patient assessment	1.0 ± 0.0	1.0 ± 0.0	7.3 ± 1.5	8.7 ± 0.6	9.0 ± 1.0	8.3 ± 1.2	5.9 ± 3.8
preparation for surgery	8.3 ± 0.6	8.0 ± 0.0	8.3 ± 0.6	8.7 ± 0.6	8.7 ± 0.6	9.7 ± 0.6	8.6 ± 0.6
Recovery and postprocedure instructions	8.3 ± 0.6	8.3 ± 0.6	8.7 ± 0.6	9.0 ± 0.0	9.0 ± 0.0	9.0 ± 0.0	8.7 ± 0.3
Procedure cost and surgeon recommendations	1.0 ± 0.0	1.0 ± 0.0	6.0 ± 0.0	8.0 ± 1.0	8.7 ± 0.6	8.7 ± 0.6	5.6 ± 3.7
Average	6.7 ± 3.5	6.6 ± 3.5	8.2 ± 1.0	8.8 ± 0.4	8.9 ± 0.2	9.3 ± 0.6	8.1 ± 2.2

Means ± standard deviations presented.

**Table 4.** Bard Performance Assessment Scores Across All Simulated Patient Inquiries (Facelift, Rhinoplasty, and Brow Lift)

Bard overall scores	Accuracy of information	Comprehensiveness	Objectivity of information	Safety of information shared with patient	Acknowledgment of limitations	Communication, clarity, patient-appropriate readability	Average
Aesthetic needs inquiry; awareness of available procedures	8.3 ± 0.6	8.7 ± 1.2	8.3 ± 0.6	8.7 ± 0.6	8.7 ± 0.6	8.7 ± 0.6	8.6 ± 0.2
Patient candidacy and procedure indications	7.3 ± 2.1	7.7 ± 1.5	7.3 ± 2.1	8.0 ± 1.0	8.0 ± 1.0	8.0 ± 1.0	7.7 ± 0.3
Procedure safety and risks	5.7 ± 1.5	3.7 ± 1.2	6.7 ± 1.2	6.7 ± 0.6	7.3 ± 0.6	8.3 ± 0.6	6.4 ± 1.6
Procedure information, steps, and techniques	7.3 ± 0.6	7.0 ± 2.0	7.7 ± 1.5	8.3 ± 0.6	8.0 ± 1.0	8.3 ± 0.6	7.8 ± 0.5
Patient assessment	1.0 ± 0.0	1.0 ± 0.0	6.0 ± 0.0	8.0 ± 1.0	9.0 ± 1.0	9.0 ± 0.0	5.7 ± 3.8
Preparation for surgery	7.3 ± 1.2	6.7 ± 3.2	8.0 ± 1.0	6.7 ± 3.2	7.7 ± 1.2	8.3 ± 1.2	7.4 ± 0.7
Recovery and postprocedure instructions	7.7 ± 0.6	7.7 ± 1.5	7.3 ± 1.2	8.0 ± 0.0	8.3 ± 0.6	8.7 ± 0.6	7.9 ± 0.5
Procedure cost and surgeon recommendations	7.3 ± 0.6	8.0 ± 1.0	6.3 ± 0.6	7.7 ± 1.2	8.0 ± 1.0	8.7 ± 0.6	7.7 ± 0.8
Average	6.5 ± 2.3	6.3 ± 2.6	7.2 ± 0.8	7.8 ± 0.7	8.1 ± 0.5	8.5 ± 0.3	7.4 ± 1.7

Means ± standard deviations presented.

**Table 5.** Comparative Relative Performance of Both Large Language Models Investigated in the Present Study, Across All Simulated Patient Inquiries (Facelift, Rhinoplasty, and Brow Lift)

Comparative analysis	Accuracy of information	Comprehensiveness	Objectivity of information	Safety of information shared with patient	Acknowledgment of limitations	Communication, clarity, patient-appropriate readability	Overall
Aesthetic needs inquiry; awareness of available procedures	ChatGPT	ChatGPT	ChatGPT	ChatGPT	ChatGPT	ChatGPT	ChatGPT
Patient candidacy and procedure indications	ChatGPT	ChatGPT	ChatGPT	ChatGPT	ChatGPT	ChatGPT	ChatGPT
Procedure safety and risks	ChatGPT	ChatGPT	ChatGPT	ChatGPT	ChatGPT	ChatGPT	ChatGPT
Procedure information, steps, and techniques	ChatGPT	ChatGPT	ChatGPT	ChatGPT	ChatGPT	ChatGPT	ChatGPT
Patient assessment	=	=	ChatGPT	ChatGPT	=	Bard	ChatGPT
Preparation for surgery	ChatGPT	ChatGPT	ChatGPT	ChatGPT	ChatGPT	ChatGPT	ChatGPT
Recovery and postprocedure instructions	ChatGPT	ChatGPT	ChatGPT	ChatGPT	ChatGPT	ChatGPT	ChatGPT
Procedure cost and surgeon recommendations	Bard	Bard	Bard	ChatGPT	ChatGPT	=	Bard
Overall	ChatGPT	ChatGPT	ChatGPT	ChatGPT	ChatGPT	ChatGPT	ChatGPT

The LLM with the higher score on each question and assessment category investigated is presented. Cases in which performance scores were identical are represented with “=.”

## DISCUSSION

The present study provides the first direct comparative analysis between ChatGPT and Bard with relevance to the potential applications of LLMs in the preoperative guidance and education of patients interested in aesthetic surgery. While Bard demonstrated significantly better baseline efficiency through its response times, ChatGPT outperformed Bard with respect to its response accuracy, comprehensiveness, objectivity, safety, and communication clarity. A statistically significant improvement in response time was also observed with ChatGPT, likely demonstrating its machine learning capabilities. Despite an inferior performance relative to ChatGPT across most question and assessment categories examined (Table 5), Bard's most notable advantage over ChatGPT represented its ability to suggest questions to “ask next” within its interactions with users—a feature that could prove useful in potentially guiding patients through informed consent.^12^ Its ability to provide surgeon recommendations based on patients’ expressed needs and geographical location represents an additional advantage over ChatGPT. Nonetheless, and despite an impressive performance by both LLMs, significant limitations persist with their use. Detailed transcripts of interactions with both LLMs are presented in Supplemental Tables 1‐6.

### Aesthetic Needs Inquiry: Awareness of Available Procedures

This first question category evaluated the LLMs’ ability to assess patients’ expressed aesthetic needs and recommend appropriate procedures. ChatGPT outperformed Bard across all 3 procedures examined within this assessment category. For the patient bothered by the loose skin and wrinkles on her face, Bard recommended facelift, brow lift, eyelid surgery, dermal fillers, or laser skin resurfacing; omitted were options including neck lift, fat grafting, toxins, dermabrasions, peels, microneedling, and radiofrequency devices. For this patient, ChatGPT's recommendations were more comprehensive, with only nonsurgical options omitted. Incorrect suggestions by Bard were observed for the patient bothered by the shape of his nose, recommending “septoplasty,” “nasal tipplasty,” “nasal bridge augmentation,” and “nasal tip reduction”—all of which are components of a rhinoplasty, without mentioning rhinoplasty itself. ChatGPT, conversely, suggested “rhinoplasty,” as well as “septoplasty,” “revision rhinoplasty,” and “nonsurgical options.” In both cases, the suggestion of septoplasty as a surgical option for a patient concerned with nasal aesthetics would be incorrect. Nonetheless, and in all cases, patients were encouraged by both LLMs to consult with a plastic surgeon who could more appropriately discuss all available options for their aesthetic needs.

### Patient Candidacy and Procedure Indications

The question within this category assessed the LLMs’ ability to comment on procedure indications and whether patients may be good candidates for specific procedures. While both ChatGPT and Bard proved capable of providing patients with general guidelines on different factors that determine patients’ candidacy for specific procedures, ChatGPT proved again to be more accurate and comprehensive in its responses. Information provided by Bard was more inaccurate and misleading at times. For example, the first criterion listed by Bard regarding a patient's candidacy for facelift was age, with a range of 30 to 60 years provided. Although age can certainly help guide patient selection, there currently exist no clinical guidelines nor cut-offs used in practice.^13^ Wherein chronology of listed indications also implies relative importance, listing age as the first criterion may be misleading to patients, by implying that it is more important than smoking status (listed third).^14,15^ Bard also listed “good skin quality” as a necessary requirement for facelift, which, while certainly associated with better outcomes, is incorrect, as poor skin quality is not a contraindication to facelift.^16^ For reference, ChatGPT listed loose skin, deep wrinkles, jowls, good general health, realistic expectations, and a nonsmoking status, demonstrating its more accurate and comprehensive performance on this section.

### Procedure Safety and Risks

Questions within this category were designed to assess the LLMs’ ability to discuss all relevant procedure risks, incidence estimates, and identify medical or lifestyle risk factors reported by patients. ChatGPT once again outperformed Bard in this question category. When queried about risks of facelift, ChatGPT effectively identified bleeding as the #1 complication, followed by scarring, nerve damage, skin discoloration, hair loss, poor wound healing, and unsatisfactory results.^14^ Nonetheless, its responses omitted seroma, sialocele, sensory changes, contour deformities, pixie ear deformity, and risk for revisions. Conversely, Bard listed infection as the first possible complication for facelift, which remains extremely rare.^17^ Impressively alluding to the use of hemostatic nets, it also listed bleeding, allergic reactions (although extremely rare), nerve damage, scarring, and “unnatural appearance,” omitting most of the relevant complications specified previously.^18^ With respect to risk estimates, ChatGPT's were found to be more accurate. Bard reported “allergic reaction to anesthesia, surgical glue, or tape” as 1% in facelift, when this should be cited as <1%, if at all.^14,18^ Bard also cited a 10% risk of “scarring,” which remains misleading and unclear. For rhinoplasty, Bard demonstrated response failure, by completely failing to provide any estimates on incidence, and for brow lift, reported a 100% risk of “pain and swelling,” which is also misleading. Bard failed to identify and counsel patients on the impact of patient-specific risk factors reported by patients (such as smoking and drug use), while, ChatGPT, for example, effectively identified and counseled the patient with Von Willebrand factor deficiency on her higher risk of bleeding during brow lift,^19^ but failed to acknowledge the increased risks associated with recreational drug use for the patient interested in rhinoplasty.^20^

### Procedure Information, Steps, and Techniques

Both LLMs proved capable of discussing the general operative steps and techniques used in the procedures of interest, although Bard proved again to be less accurate and comprehensive. ChatGPT provided organized step-by-step explanations of relevant surgical steps at patient-appropriate levels. For facelift, it explained differences between the traditional facelift, mini facelift, deep plane facelift, and SMAS (Superficial Musculoaponeurotic System) plication/imbrication, although its definition of a composite facelift^21^ was inaccurate, equating it to the “lift and fill” facelift.^22,23^ In contrast, Bard reported on only the subcutaneous facelift, SMAS facelift, and deep plane facelift. For rhinoplasty, Bard discussed open rhinoplasty, closed rhinoplasty, septoplasty, “tipplasty,” bridge augmentation, and tip reduction. Although ChatGPT's responses were noted again to be superior and more patient appropriate for rhinoplasty and brow lift, both models’ responses remained incomprehensive; for example, failing to discuss dorsal preservation techniques,^24^ or direct brow lift.^25^ Additionally, for brow lift, both models failed to emphasize that the traditional coronal approach has fallen out of favor to endoscopic techniques.^26^

### Patient Assessment

This question was designed to evaluate the LLMs’ ability to analyze images submitted by patients, perform an aesthetic evaluation, and suggest appropriate techniques or procedures. Both models demonstrated complete response failures in this section. Given that ChatGPT-3, the free version of the model readily available to patients, was used in this study, it lacks computer vision capabilities,^27^ and thus, its response failures on this section were expected. Nonetheless, it educated patients on considerations used by plastic surgeons in the requested assessment, such as skin elasticity and ptosis in facelift, or bridge shape and nasal skin thickness in rhinoplasty, for example. Similarly, Bard claimed to be unable to analyze images. Deferring to plastic surgeons for the requested assessment, Bard was also inconsistent in its ability to educate patients on factors surgeons may use in this context. It discussed considerations such as the extent of forehead skin, and location of a patient's hairline used by surgeons during the assessment for brow lift, for example, but failed to provide any insight in this regard for the patients interested in facelift or rhinoplasty.

### Preparation for Surgery

Questions within this category assessed the LLMs’ ability to explain the preoperative process in the lead up to surgery, suggest ways for patients to prepare, and counsel patients on necessary lifestyle modifications to improve their outcomes. ChatGPT again outperformed Bard across all 3 procedures examined. Bard was capable of providing a general overview of the preoperative consultation process, discussing elements such as the initial consultation and preoperative investigations. While discussing the need for smoking cessation and holding anticoagulation, responses were noted to be general and not individualized, using terms like “if you smoke, you will need to stop smoking…,” despite the patient already reporting being an active smoker. For the patient with a Von Willebrand factor deficiency interested in brow lift, Bard missed the opportunity to emphasize the need for consultation with a hematologist, whereas ChatGPT emphasized this point. Some of Bard's suggestions were also unsafe and blatantly incorrect, for example, suggesting to the patient seeking information on brow lift to eat “a healthy breakfast on the morning of your surgery.” Overall, and with Bard acknowledging its limitations as an AI model significantly fewer times in its responses than ChatGPT (62% vs 95%, respectively, *P* < .001), Bard's performance on this section accentuates the importance of qualifying all medical recommendations to patients with these disclaimers. In contrast, ChatGPT provided more organized, safe, and structured responses, discussing important elements in the preoperative process that were omitted by Bard, such as informed consent, preoperative anesthesia consultations, and financial arrangements.

### Recovery and Postprocedural Instructions

Questions within this category solicited the models’ ability to explain the recovery process, discuss relevant activity limitations following surgery, and time before which patients may return to work. Once again, ChatGPT outperformed Bard within this question category. Bard suggested that a full recovery from facelift can be expected to occur within 4 to 6 weeks following surgery, but that “it may take up to 12 months for the final results to be visible.” It suggested that 4 to 6 weeks are necessary following brow lift before being able to return to work, and omitted critical information on postoperative recovery expectations in rhinoplasty, such as nasal congestion. It suggested that the facelift patient finds someone who could help her wash her hair within the first few days following surgery, despite water exposure to incisions immediately postoperatively being not advisable. The timelines it provided to patients regarding required assistance in daily activities postoperatively were also more general and ambiguous compared with those provided by ChatGPT, using terms like “first few days” or “few weeks” rather than giving more specific estimates.

ChatGPT's responses were much more organized and comprehensive. It educated patients on expectations during recovery across the entire postoperative period, from immediately postoperatively in the postanesthesia care unit, to a few days, first week, first month, and a long-term recovery following surgery. It provided more specific and explicit date ranges for expected activity limitations and required assistance, as well as times before which normal activities and work can be resumed. It accurately counseled patients on expected challenges with postoperative recovery such as pain, swelling, and bruising, and suggested strategies, such as head elevation and cold compresses, to help mitigate them.

### Procedure Cost and Surgeon Recommendations

This question was designed to assess the LLMs’ ability to provide patients with estimates on procedure costs, and suggest qualified surgeons within the patient's geographic location. This category was the only one in which Bard outperformed ChatGPT. ChatGPT exhibited response failures with an inability to provide patients with estimates on the costs of any of the procedures examined, nor recommend local surgeons. Nonetheless, it suggested strategies to help find surgeons through online searches, recommendations from healthcare professionals, friends, and family, or professional organizations such as the American Society of Plastic Surgeons. It encouraged patients to consider surgeon qualifications, experience, certifications, patient reviews, as well as before-and-after pictures, while also recommending consultations with multiple surgeons before choosing one.

Bard, in contrast, was capable of providing reasonable price estimates for facelift, rhinoplasty, and brow lift, discussing strategies to help pick the right surgeon based on experience, training, results, pricing, and communication, and provided direct surgeon recommendations based on patient-reported geographical locations. However, it remains unclear how these recommendations were made. All 3 surgeons recommended for facelift were board certified in plastic surgery, whereas for rhinoplasty, all surgeons recommended were “facial plastic surgeons,” board certified in head and neck surgery, and not plastic surgery. For brow lift, surgeons recommended included 2 head and neck surgeons, 1 plastic surgeon (who focuses mainly on breast), whereas many other surgeons, including the study's senior author, for example, were not included. It remains interesting to investigate how Bard arrives at these recommendations, as this could represent a novel target for patient outreach and marketing. Interestingly, recommended surgeons did not represent the same ones appearing as “top hits” when the same Google search is performed, indicating that the algorithm used by Google for online searches and Bard are different.

### Sources and Evidence-Based Recommendations

The evidence-based nature of responses and recommendations provided to patients by LLMs remains elusive. Many instances of incorrect recommendations were identified, as discussed previously, while other recommendations, albeit not necessarily incorrect, lacked supporting evidence. For example, Bard suggested that a patient stay hydrated in the weeks leading up to surgery, in order to “reduce the risk of complications.” While not a dangerous suggestion, this recommendation lacks evidence and can contribute to increased patient anxiety in the preoperative period. While ChatGPT failed to provide any references in its responses, Bard provided sources for only 2 of its responses (5%), both of which representing webpages of plastic surgery practices, rather than scientific evidence. This is unsurprising given that LLMs are trained on massive amounts of data across the breadth of the Internet.^1^ However, with emerging AI technology such as scite (Scite, Brooklyn, New York, NY) with access to scientific literature, and further development of these LLMs, it is expected that responses could grow to become more based off scientific evidence, and thus of greater value to clinical applications in aesthetic surgery.

### Potential Applications and Clinical Relevance

The present study sheds light on the current and future applications of AI LLM technology for the preoperative guidance and education of patients interested in aesthetic surgery. With patients increasingly turning to the Internet for their self-directed research about their medical and aesthetic needs, the present study provides surgeons with a detailed and objective assessment of the quality of the information currently shared by LLM models with our patients. Additionally, for plastic surgeons, the present study helps identify ChatGPT as the superior LLM that could be used as a basis for a new, tailored, and fine-tuned AI model, trained on evidence-based information selected and curated by our professional societies, and tailored to surgeon-specific preferences, to which patients may be directed in the preoperative period. “This would represent a response by our specialty to these evolving patient resources, providing patients with interactive and patient-specific educational references, in contrast to pamphlets and websites currently used, which patients may use to become better informed about procedures of interest to them in the lead up to surgery.”

### Limitations

The present study is not without its limitations. The senior author's evaluation of performance nonetheless represents a single surgeon's perspective. Aesthetic surgery remains personalized and nuanced, wherein questions posed by patients may have multiple subjectively correct answers. Nonetheless, by evaluating the responses of both LLMs, side by side, using the objective assessment tool of AI performance developed for the present study, a relative assessment and comparison of performance is achieved for both LLMs in the investigated application. While ChatGPT-3 was used in the present study as it represents the freely available version of the technology readily accessible by patients, it remains unclear how the performance of its latest version, ChatGPT-4, would differ in this regard. Furthermore, given the rapidly evolving nature of this technology, it may be that freely available models may meet or surpass the capabilities of ChatGPT-4 in the future. Finally, LLMs are an amalgam of information on which they are trained; the impact of the evolving information on which these LLMs are trained remains to be established, as is the reliability and reproducibility of their medical recommendations. These considerations remain subjects of ongoing studies by our group.

## CONCLUSIONS

The present study demonstrates that LLMs represent developing resources for the preoperative guidance and education of patients interested in aesthetic surgery, with imperfect performance at this time. ChatGPT (OpenAI, San Francisco, CA) outperformed Bard (Google, Mountain View, CA) in all assessment categories examined, with more accurate, comprehensive, objective, safe, and clear responses provided. Bard's response times were significantly faster than ChatGPT's, although ChatGPT, but not Bard, demonstrated machine learning through significant improvements in response times. ChatGPT more readily acknowledged its limitations as an AI model and encouraged consultation with plastic surgeons, whereas both models failed to qualify any of their responses with scientific evidence. The present study identifies a need for further development, refinement, and evidence-based qualification of information provided to patients by LLMs before their safe and effective implementation in aesthetic surgery.

## Supplemental Material

This article contains supplemental material located online at www.asjopenforum.com.

## Supplementary Material

ojae058_Supplementary_Data
