# Supplementary material for: Comparative Performance of Current Patient-Accessible Artificial Intelligence Large Language Models in the Preoperative Education of Patients in Facial Aesthetic Surgery
Source: Aesthet Surg J Open Forum. 2024 Aug 13;6:ojae058. doi: 10.1093/asjof/ojae058 (PMC11371156; doi:10.1093/asjof/ojae058)
Supplement: ojae058_Supplementary_Data [file ojae058_supplementary_data.docx]

**Supplementary Table 1: ChatGPT Performance Assessment Scores for the Simulated Patient Inquiry into Facelift**

| **ChatGPT:**  **Facelift** | Accuracy of Information | Comprehensiveness | Objectivity of Information | Safety of Information Shared with Patient | Acknowledgement of Limitations | Communication, Clarity, Patient-Appropriate Readability | **Average** |
| --- | --- | --- | --- | --- | --- | --- | --- |
| Aesthetic Needs Inquiry; Awareness of Available Procedures | 9 | 8 | 9 | 9 | 9 | 10 | 9 |
| Patient Candidacy and Procedure Indications | 9 | 10 | 9 | 10 | 9 | 10 | 9.5 |
| Procedure Safety & Risks | 8 | 7 | 8 | 9 | 9 | 9 | 8.3 |
| Procedure Information, Steps & Techniques | 8 | 9 | 9 | 9 | 9 | 9 | 8.8 |
| Patient Assessment | 1 | 1 | 9 | 9 | 10 | 7 | 6.2 |
| Preparation for Surgery | 8 | 8 | 8 | 8 | 8 | 9 | 8.2 |
| Recovery & Post-Procedure Instructions | 8 | 8 | 8 | 9 | 9 | 9 | 8.5 |
| Procedure Cost & Surgeon Recommendations | 1 | 1 | 6 | 8 | 8 | 8 | 5.3 |
| **Average** | 6.5 | 6.5 | 8.3 | 8.9 | 8.9 | 8.9 | 8.0 |

**Supplementary Table 2: ChatGPT Performance Assessment Scores for the Simulated Patient Inquiry into Rhinoplasty**

| **ChatGPT:**  **Rhinoplasty** | Accuracy of Information | Comprehensiveness | Objectivity of Information | Safety of Information Shared with Patient | Acknowledgement of Limitations | Communication, Clarity, Patient-Appropriate Readability | **Average** |
| --- | --- | --- | --- | --- | --- | --- | --- |
| Aesthetic Needs Inquiry; Awareness of Available Procedures | 9 | 10 | 9 | 9 | 9 | 10 | 9.3 |
| Patient Candidacy and Procedure Indications | 9 | 9 | 9 | 9 | 9 | 10 | 9.2 |
| Procedure Safety & Risks | 8 | 8 | 8 | 8 | 8 | 9 | 8.2 |
| Procedure Information, Steps & Techniques | 9 | 8 | 8 | 9 | 9 | 10 | 8.8 |
| Patient Assessment | 1 | 1 | 7 | 9 | 9 | 9 | 6.0 |
| Preparation for Surgery | 9 | 8 | 9 | 9 | 9 | 10 | 9.0 |
| Recovery & Post-Procedure Instructions | 9 | 9 | 9 | 9 | 9 | 9 | 9.0 |
| Procedure Cost & Surgeon Recommendations | 1 | 1 | 6 | 9 | 9 | 9 | 5.8 |
| **Average** | 6.9 | 6.8 | 8.1 | 8.9 | 8.9 | 9.5 | 8.2 |

**Supplementary Table 3: ChatGPT Performance Assessment Scores for the Simulated Patient Inquiry into Brow Lift**

| **ChatGPT:**  **Brow Lift** | Accuracy of Information | Comprehensiveness | Objectivity of Information | Safety of Information Shared with Patient | Acknowledgement of Limitations | Communication, Clarity, Patient-Appropriate Readability | **Average** |
| --- | --- | --- | --- | --- | --- | --- | --- |
| Aesthetic Needs Inquiry; Awareness of Available Procedures | 9 | 9 | 9 | 9 | 9 | 10 | 9.2 |
| Patient Candidacy and Procedure Indications | 9 | 9 | 9 | 9 | 9 | 10 | 9.2 |
| Procedure Safety & Risks | 9 | 9 | 9 | 9 | 9 | 9 | 9.0 |
| Procedure Information, Steps & Techniques | 8 | 7 | 9 | 9 | 9 | 9 | 8.5 |
| Patient Assessment | 1 | 1 | 6 | 8 | 8 | 9 | 5.5 |
| Preparation for Surgery | 8 | 8 | 8 | 9 | 9 | 10 | 8.7 |
| Recovery & Post-Procedure Instructions | 8 | 8 | 9 | 9 | 9 | 9 | 8.7 |
| Procedure Cost & Surgeon Recommendations | 1 | 1 | 6 | 7 | 9 | 9 | 5.5 |
| **Average** | 6.6 | 6.5 | 8.1 | 8.6 | 8.9 | 9.4 | 8.0 |

**Supplementary Table 4: Bard Performance Assessment Scores for the Simulated Patient Inquiry into Facelift**

| **Bard:**  **Facelift** | Accuracy of Information | Comprehensiveness | Objectivity of Information | Safety of Information Shared with Patient | Acknowledgement of Limitations | Communication, Clarity, Patient-Appropriate Readability | **Average** |
| --- | --- | --- | --- | --- | --- | --- | --- |
| Aesthetic Needs Inquiry; Awareness of Available Procedures | 8 | 8 | 8 | 9 | 9 | 9 | 8.5 |
| Patient Candidacy and Procedure Indications | 5 | 6 | 5 | 7 | 7 | 7 | 6.2 |
| Procedure Safety & Risks | 6 | 3 | 6 | 7 | 7 | 8 | 6.2 |
| Procedure Information, Steps & Techniques | 7 | 5 | 6 | 8 | 7 | 8 | 6.8 |
| Patient Assessment | 1 | 1 | 6 | 7 | 10 | 9 | 5.7 |
| Preparation for Surgery | 8 | 8 | 8 | 8 | 7 | 9 | 8.0 |
| Recovery & Post-Procedure Instructions | 8 | 9 | 8 | 8 | 8 | 9 | 8.3 |
| Procedure Cost & Surgeon Recommendations | 8 | 8 | 6 | 7 | 8 | 9 | 7.7 |
| **Average** | 6.4 | 6.0 | 6.6 | 7.6 | 7.9 | 8.5 | 7.2 |

**Supplementary Table 5: Bard Performance Assessment Scores for the Simulated Patient Inquiry into Rhinoplasty**

| **Bard:**  **Rhinoplasty** | Accuracy of Information | Comprehensiveness | Objectivity of Information | Safety of Information Shared with Patient | Acknowledgement of Limitations | Communication, Clarity, Patient-Appropriate Readability | **Average** |
| --- | --- | --- | --- | --- | --- | --- | --- |
| Aesthetic Needs Inquiry; Awareness of Available Procedures | 9 | 10 | 9 | 9 | 9 | 9 | 9.2 |
| Patient Candidacy and Procedure Indications | 8 | 8 | 8 | 8 | 8 | 8 | 8.0 |
| Procedure Safety & Risks | 7 | 3 | 8 | 6 | 8 | 9 | 6.8 |
| Procedure Information, Steps & Techniques | 8 | 7 | 8 | 8 | 8 | 8 | 7.8 |
| Patient Assessment | 1 | 1 | 6 | 9 | 9 | 9 | 5.8 |
| Preparation for Surgery | 8 | 9 | 9 | 9 | 9 | 9 | 8.8 |
| Recovery & Post-Procedure Instructions | 7 | 6 | 6 | 8 | 8 | 8 | 7.2 |
| Procedure Cost & Surgeon Recommendations | 7 | 9 | 6 | 9 | 9 | 9 | 8.2 |
| **Average** | 6.9 | 6.6 | 7.5 | 8.3 | 8.5 | 8.6 | 7.7 |

**Supplementary Table 6: Bard Performance Assessment Scores for the Simulated Patient Inquiry into Brow Lift**

| **Bard:**  **Brow Lift** | Accuracy of Information | Comprehensiveness | Objectivity of Information | Safety of Information Shared with Patient | Acknowledgement of Limitations | Communication, Clarity, Patient-Appropriate Readability | **Average** |
| --- | --- | --- | --- | --- | --- | --- | --- |
| Aesthetic Needs Inquiry; Awareness of Available Procedures | 8 | 8 | 8 | 8 | 8 | 8 | 8.0 |
| Patient Candidacy and Procedure Indications | 9 | 9 | 9 | 9 | 9 | 9 | 9.0 |
| Procedure Safety & Risks | 4 | 5 | 6 | 7 | 7 | 8 | 6.2 |
| Procedure Information, Steps & Techniques | 7 | 9 | 9 | 9 | 9 | 9 | 8.7 |
| Patient Assessment | 1 | 1 | 6 | 8 | 8 | 9 | 5.5 |
| Preparation for Surgery | 6 | 3 | 7 | 3 | 7 | 7 | 5.5 |
| Recovery & Post-Procedure Instructions | 8 | 8 | 8 | 8 | 9 | 9 | 8.3 |
| Procedure Cost & Surgeon Recommendations | 7 | 7 | 7 | 7 | 7 | 8 | 7.2 |
| **Average** | 6.3 | 6.3 | 7.5 | 7.4 | 8.0 | 8.4 | 7.3 |
